# Effects of Barium Concentration on Oropharyngeal Swallow Timing Measures

**DOI:** 10.1007/s00455-013-9485-6

**Published:** 2013-09-18

**Authors:** Shauna L. Stokely, Sonja M. Molfenter, Catriona M. Steele

**Affiliations:** 1 Toronto Rehabilitation Institute, University Health Network, 550 University Avenue, #12-125, Toronto, ON M5G 2A2 Canada; 2 Department of Speech Language Pathology, University of Toronto, Toronto, ON Canada; 3 Bloorview Research Institute, Holland Bloorview Kids Rehabilitation Hospital, Toronto, ON Canada

**Keywords:** Dysphagia, Deglutition, Deglutition disorders, Videofluoroscopy, Barium concentration

## Abstract

Videofluoroscopy is commonly used for evaluating oropharyngeal swallowing but requires radiopaque contrast (typically barium). Prior studies suggest that some aspects of swallowing, including timing measures of oral and pharyngeal bolus transit, vary depending on barium concentration. The aim of our study was to identify timing differences in healthy swallowing between “thin” (40 % w/v concentration) and “ultrathin” (22 % w/v concentration) barium solutions. Twenty healthy adults (Ten women; mean age = 31 years) each performed a series of three noncued 5-ml swallows each of ultrathin and thin liquid barium solutions in videofluoroscopy. Timing measures were compared between barium concentrations using a mixed-model ANOVA. The measures of interest were stage transition duration, pharyngeal transit time, and duration of upper esophageal sphincter opening. Significant differences were observed in the timing measures of swallowing with respect to barium concentration. In all cases, longer durations were seen with the higher barium concentration. Barium concentration influences timing parameters in healthy swallowing, even between ultrathin and thin concentrations. Clinicians need to understand and control for the impact of different barium stimuli on swallowing physiology.

Videofluoroscopic swallow studies (VFSS) are a commonly used instrumental method for evaluating oropharyngeal safety for and efficiency of individuals with suspected dysphagia (swallowing impairment) [1]. In order to view a bolus under fluoroscopy, all food and stimuli must contain a radiopaque element. In North America, barium sulfate is widely used as this radiopaque element. Some commercially available products (e.g., Varibar^®^) containing a controlled concentration of barium sulfate are offered in a range of consistencies (e.g., thin, nectar-thick, honey-thick); however, these are not universally used nor are they even available in some regions outside the United States. For institutions without access to these products, clinicians have no alternative but to prepare their own VFSS stimuli by mixing barium sulfate liquid or powder with regular liquids and solids to achieve radiopacity. One survey of Canadian clinicians practicing in dysphagia indicated that it was not common to use standardized protocols for preparing in-house stimuli [1]. The use of nonstandardized barium stimuli is concerning because of the likelihood that uncontrolled variation in physical properties of the bolus, such as viscosity, may influence swallowing physiology [2, 3].

A primary goal of the VFSS is to determine what textures the patient can safely consume; however, the addition of barium sulfate to liquids used as VFSS stimuli changes the density and the viscosity of the original liquid [4–6]. Changes in viscosity are dependent on the concentration of barium in the solution: Dantas et al. [7] reported that a 1.40-g/ml thin-liquid solution (equivalent to a 140 % w/v solution), made with E-Z-HD barium sulfate and distilled water, had a viscosity of 200 cP, but that a 2.50-g/ml thin-liquid solution (equivalent to a 250 % w/v solution) made with the same powder had a viscosity of 300 cP. Water without any barium added would have a viscosity of approximately 1 cP at room temperature and a density of 1 g/ml [8]. Previous research has shown that even small viscosity differences, such as that between orange juice and tomato juice, can have functional effects on a patient’s swallowing safety [9]. If the viscosity changes that occur as the result of adding barium are enough to alter an individual’s typical swallow, then VFSS findings may not be representative of typical swallowing behavior and would therefore lack construct validity. Thus, it is important to consider the effects of differences in barium concentration on swallowing.

This area was previously investigated by Dantas et al. [7], who looked at how barium concentration affected the durations of the oral and pharyngeal phases of the swallow in healthy young male participants. They used the term density to refer to the relative concentration of barium in the two stimuli used in their study: either “high density” (2.50 g/ml) or “low density” (1.40 g/ml). For both 5- and 10-ml volumes of these stimuli, the results showed significantly longer oral and pharyngeal transit times with the higher concentration solution. Additionally, the upper esophageal sphincter (UES) was open longer with both volumes of the higher concentration solution. This evidence shows that the concentration of barium in a liquid solution affects temporal aspects of healthy swallowing. However, the stimuli used in the Dantas study [7] involved barium concentrations that are higher than those typically used in VFSS. Given that the smallest magnitude of viscosity change that causes a significant effect in swallowing behaviors has not yet been determined [10], it is of interest to determine whether similar trends would be observed in comparisons of lower-concentration barium solutions.

There is very little information available in the scientific literature describing differences in swallowing between low-concentration barium solutions. Fink and Ross [11] studied aspiration rates related to barium concentration in a group of 40 patients referred for VFSS. Those patients who did not aspirate on initial trials of a thin 40 % w/v solution (full-strength Varibar^®^) were given an ultrathin solution of ~22 % w/v concentration, prepared by diluting thin-liquid Varibar^®^ in a 50:50 ratio with water. Note that the concentration of the diluted solution used by Fink and Ross is estimated to have been ~22 % w/v rather than 20 % w/v (i.e., slightly more than half the concentration of full-strength Varibar^®^), based on the molecular weight and weight-to-weight density of the barium in this recipe. The results showed that half of the participants who were deemed safe (i.e., no aspiration) with the 40 % w/v solution aspirated with the lower-concentration solution. Based on this finding, the authors argued that an ~22 % w/v barium sulfate solution behaved more like water and was closer to a “true thin liquid” than the 40 % w/v solution. What has yet to be determined are potential mechanisms that explain this difference, such as the trend discovered by Dantas et al. [7] that lower-concentration barium solutions travel faster through the oropharynx. The purpose of the present study was to address this gap in the literature. The specific question that we set out to address was: In healthy participants, when volume is held constant, do stage transition duration, pharyngeal transit time, and duration of UES opening vary as a function of barium concentration? Based on the previous research by Dantas et al. [7] and Fink and Ross [11], we hypothesized that all three of these timing measures would be significantly shorter with a 22 % w/v solution than with a 40 % w/v solution.

## Methods

### Participants

Twenty healthy participants (Ten males) between 22 and 45 years of age (mean = 31.5 years, standard deviation = 5.7 years) completed the protocol. Participants had no history of swallowing difficulty, neurological deficits, or head or neck surgery (apart from routine dental surgery, tonsillectomy, or adenoidectomy). All participants voluntarily consented to be in the study, which was approved by the institution’s research ethics board.

### Radiographic Procedure

Participants were seated in a lateral view for the VFSS exam. The fluoroscope was positioned for the following boundaries to be visible on the images: lips anteriorly, posterior pharyngeal wall posteriorly, cervical esophagus inferiorly, and nasopharynx superiorly. Fluoroscopy was conducted using a Toshiba Ultimax fluoroscope (Toshiba America Medical Systems Inc., Tustin, CA) at 30 pulses per second, with images captured at 30 frames per second using the Digital Swallowing Workstation (KayPentax, Lincoln Park, NJ). A certified and licensed speech-language pathologist conducted every VFSS, with the assistance of a radiation technologist.

### Stimuli

Participants drank three self-administered 5-ml boluses of 40 % w/v concentration barium solution and three 5-ml boluses of 22 % w/v concentration barium solution, both made with Liquid Polibar^®^, a 100 % w/v barium suspension, which was diluted to the experimental concentrations with water. The boluses were self-administered from a medicine cup; the participants were instructed to “drink all the liquid [in the cup] in one sip as normally as possible.” Each cup was weighed before and after the sip to calculate the exact amount of liquid consumed. The order of presentation was randomized by condition so that participants either completed the three 40 % w/v swallows followed by the three 22 % w/v swallows or the vice versa.

### Data Processing

The VFSS recordings were segmented into individual anonymized bolus clips. Frame-by-frame analysis was performed using Corel Video Studio Pro X4 by two trained research assistants who were blinded to each other’s ratings as well as to the research questions and hypotheses. Prior to analyzing the actual data, the research assistants were taught by a speech-language pathologist to accurately find the following frames of interest for each swallow recording:

- the first frame showing the bolus head crossing the ramus of the mandible;
- the frame showing the “jump” of the hyoid, as indicated by marked anterior–superior movement of the hyoid bone;
- the frame showing the onset of UES opening;
- the frame showing the UES closing (simultaneous with the tail of the bolus passing through the UES).

These events were then used to derive the following timing measures:

- Stage transition duration (STD) (in ms): the interval between the bolus head crossing the ramus of the mandible and the onset of hyoid elevation;
- Pharyngeal transit time (PTT) (in ms): the interval between the bolus head crossing the ramus of the mandible and closing of the UES;
- Duration of UES opening (in ms): interval from the initial opening of the UES until it is fully closed.

Interrater reliability was tested for each parameter on a random selection of 10 % of the swallows using two-way mixed intraclass coefficients (ICC) for consistency. ICCs (with 95 % confidence intervals) were 0.9 (0.67–0.97) for UES opening duration, 0.98 (0.92–0.99) for pharyngeal transit time, and 0.99 (0.97–1.0) for stage transition duration. Intrarater reliability was calculated similarly with ICCs of 0.82 (0.59–0.92) for UES opening duration, 0.97 (0.93–0.99) for pharyngeal transit time, and 0.97 (0.92–0.99) for stage transition duration. These measures show excellent agreement for all measures [12], with the lower confidence interval boundaries for UES opening duration measures falling in the “good” range. The lower agreement for identifying UES opening and closing events is not entirely unexpected, given that the UES spans a region from C4 to C6 and that it moves upward during swallowing, making the identification of the boundaries of the sphincter slightly more subjective.

### Statistical Analysis

Fully factorial mixed-model analyses of variance were used to determine whether differences in temporal measures of swallowing were present between the 22 and 40 % w/v concentrations of barium. This analysis was conducted with within-participant factors of barium concentration and swallow number, and an α criterion of *p* < 0.05 for statistical significance. A compound symmetry structure was found to have the best model fit. Cohen’s *d* effect size measures were used to determine the strength of any statistically significant findings. This measure compares the group mean difference as a ratio of the pooled standard deviation; values from 0.2 to 0.5 reflect small effect size, values between 0.51 and 0.8 reflect medium effect size, and values greater than 0.8 reflect a large effect size [13].

## Results

Descriptive statistics (means and 95 % confidence intervals) for the temporal parameters of interest are summarized by barium concentration in Table 1. For all three measures of interest, durations were significantly longer for the 40 % w/v solution than for the 22 % w/v solution. The following sections provide further information regarding these parameters.

**Table 1:** Descriptive statistics for temporal measures with thin and ultrathin solutions

Parameter (ms)	22 % w/v		40 % w/v		Statistical significance	Effect size
	Mean	95 % CI	Mean	95 %	*p* value	Cohen’s *d*
Stage transition duration	33	−30 to 96	94	31–157	0.01	0.38 (small)
Pharyngeal transit time	464	406–541	678	611–745	0.000	1.02 (large)
UES opening duration	282	242–323	408	368–449	0.000	0.99 (large)

### Stage Transition Duration (STD)

Significantly longer stage transition durations were seen with the 40 % w/v solution than with the 22 % w/v solution, *F*(1, 93.894) = 6.994, *p* = 0.01, Cohen’s *d* = 0.38, i.e., a small effect size. Negative values in stage transition duration, such as those observed for the 22 % w/v solution, indicate that the onset of hyoid elevation occurred prior to the bolus head crossing the ramus of the mandible.

### Pharyngeal Transit Time (PTT)

Significantly longer pharyngeal transit times were observed with the 40 % w/v solution than with the 22 % w/v solution, *F*(1,93.916) = 61.412, *p* = 0.000. For this result, the effect size was much larger (*d* = 1.02).

### Duration of UES Opening

Finally, significantly longer durations of UES opening were observed with the 40 % w/v solution than with the 22 % w/v solution, *F*(1,94.139) = 62.867, *p* = 0.000. As with the previous result, the effect size was large (*d* = 0.99).

## Discussion

The purpose of this study was to determine whether temporal differences seen when comparing high-concentration barium solutions [7] would persist when comparing low-concentration barium solutions, such as those more commonly used in clinical practice (i.e., 40 and 22 % w/v). The results of this study are clear: for all temporal measures of interest (i.e., stage transition duration, pharyngeal transit time, and duration of UES opening), significantly shorter durations were seen with the 22 % w/v barium sulfate solution than with the 40 % w/v solution. The differences observed in pharyngeal transit time and UES opening duration were particularly marked, with large effect sizes. These results concur with previous findings that indicate barium concentration in liquid stimuli affects temporal events in healthy swallowing and that solutions with a lower concentration of barium elicit shorter event durations [7]. These results also support our hypothesis that even small differences in barium concentration have an impact on swallowing. Lastly, this study supports the suggestion that a 22 % w/v “ultrathin” solution may act more like a true thin fluid such as water than a 40 % w/v solution [11]. Although lower concentrations of barium appear less opaque on fluoroscopy, the study by Fink and Ross [11], together with our own use of a 22 % w/v concentration for several years, suggests that this concentration is adequate for visualization.

An important consideration when exploring temporal measures in healthy participants is the degree of variability that has previously been observed in studies of healthy swallowing. Molfenter and Steele [14] reviewed existing data for the six most commonly reported temporal measures in the literature. Using modified forest plots to chart measures of central tendency and spread, they showed that some measures, like the duration of UES opening and the duration of the laryngeal closure-to-UES opening interval, displayed little variability. Other measures, such as STD and pharyngeal transit time, were more variable with wider confidence intervals. Previously reported trends with respect to normal variation in these measures suggest that the significant difference and large effect size seen in UES opening duration in this study is a particularly notable finding, given that this measure typically has tight values.

This study has several implications for clinical practice. First, swallowing behaviors (as measured using temporal parameters) can be expected to vary, based solely on changing the concentration of barium in a test liquid. This highlights the need for clinicians to know what concentration of barium they are using and to have an understanding of how the concentration of barium in VFSS stimuli will affect their patient’s swallowing physiology. For example, if a clinician uses a more concentrated solution, longer transit times may be wholly or partly attributable to barium concentration rather than indicative of impairment. Second, thin-liquid barium stimuli should be prepared according to the manufacturer’s instructions to achieve the intended concentrations for use, and if off-label concentrations are desired, as is the case when preparing “ultrathin” barium [11], standardized recipes should be followed. This principle should also be applied to VFSS research. Finally, we echo the suggestion made by Cichero and colleagues [5] that it is necessary to objectively measure opaque liquids to better understand rheological differences between test stimuli and their nonopaque counterparts [15]. With increased understanding, the ultimate goal will be to match the viscosity and other rheological properties of stimuli used in VFSS with the texture and thickness of the food and liquid that is served to patients with dysphagia. In doing so, the validity and generalizability of the VFSS procedure will be strengthened. Moving forward, it will be important to determine whether barium concentration also influences timing measures with thicker bolus consistencies, including nectar, honey, and spoon-thick liquids. Such data will enable clinicians to develop realistic expectations for VFSS and will help to elucidate features of swallowing that are attributable to barium concentration as opposed to pathophysiology.
